# Physical activity and weight are important predictors of health related quality of life in adults with congenital heart disease

**DOI:** 10.1016/j.ijcchd.2025.100588

**Published:** 2025-04-25

**Authors:** Constance G. Weismann, Frishta Jafar, Joanna Hlebowicz

**Affiliations:** a Department of Clinical Sciences Lund, Lund University, Sweden; b Department of Pediatric Cardiology, Skåne University Hospital, Lund, Sweden; c Department of Pediatric Cardiology and Pediatric Intensive Care, Ludwig Maximilium University, Munich, Germany; d Department of Cardiology, Skåne University Hospital, Lund, Sweden

## Abstract

**Introduction:**

Traditional cardiovascular risk factors put patients with congenital heart disease (CHD) at increased risk for acquired cardiovascular disease and mortality – more so than patients without CHD. In the general population, health related quality of life (HRQoL) is associated with regular physical activity. It was the aim of this study to evaluate the most important predictors of HRQoL in adults with CHD (ACHD).

**Methods:**

This is a registry study using single center data collected between 2004 and 2022. Data include demographic data such as age and sex, body mass index (BMI) type of CHD, prior surgeries, physical activity and HRQoL using the EQ-5D-3L questionnaire. CHD severity was classified based on European Society of Cardiology (ESC) criteria. The cohort was divided based on self-reported levels of physical activity.

**Results:**

A total of 2469 patients were included in this study. 878 (25.6 %) patients had mild, 1151 (46.9 %) moderate and 329 (13.3 %) severe CHD. Patients with severe CHD had a lower BMI, HRQoL and were less physically active than those with mild-moderate CHD. Conversely, patients who were not doing regular exercise were significantly older, were more likely to be female, had a higher BMI, and had a lower HRQoL than their physically active peers. In a logistic regression model, physical activity was the most important predictor of a perfect HRQoL score in all five domains, especially if performed for ≥3 h/week (Odds ratios (OR) 2.1–7.5, all p < 0.001). In patients with severe CHD, HRQoL was disproportionately increased with even little exercise. Other important predictors of perfect HRQoL were younger age (OR 0.99, p < 0.001), male sex (OR 1.58, p < 0.001), mild-moderate CHD (OR 1.59, p < 0.001) and being of normal/overweight (OR 1.44, p < 0.001). Patients with underweight or obesity had a higher HRQoL only if execrising ≥3 h/week.

**Conclusion:**

Regular physical activity in ACHD patients is associated with better HRQoL. Patients with underweight and obesity alike are also at risk for impaired HRQoL. We suggest that ACHD follow-up visits should include counseling on life-style issues in order to enhance HRQoL and minimize modifiable risk factors for acquired cardiovascular disease.

## Highlights

• Physical activity is the most important predictor of a perfect HRQoL score in adults with CHD.
• In patients severe CHD, HRQoL is disproportionately increased with only some physical, i.e. <3 h/week.
• Adult CHD patients with underweight and obesity alike are at increased risk for impaired HRQoL. Execrising ≥3 h/week is associated with HRQoL only in this cohort.
• Non-modifiable predictors of HRQoL are younger age, male sex, and mild-moderate CHD.

## Graphical abstract

**Figure:**
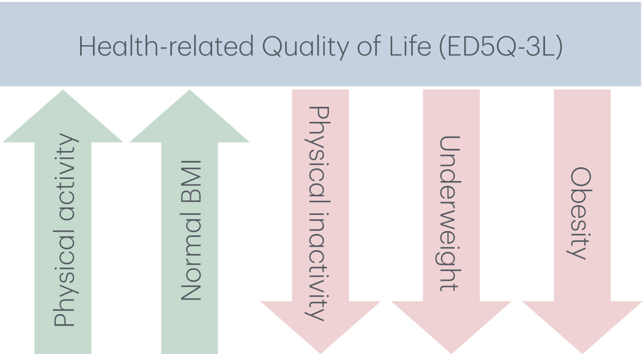

## 1 Introduction

The population of adults with congenital heart disease (ACHD) has been growing continuously for decades due to improving treatment options. In Sweden, 97 % of individuals born with congenital heart disease (CHD) reach adulthood [1]. In the current era, ACHD outnumber children in every type of CHD, making other factors such as risk for acquired cardiovascular disease and health related quality of life (HRQoL) increasingly more important [2]. ACHD with mild to moderate defects have been identified to have an elevated risk for acquired cardiovascular disease, one that is uncharacteristically high even when adjusting for other additional risk factors [3]. By contrast, HRQoL appears to be mostly impaired among ACHD patients with severe disease complexity [4,5]. Specifically, physical and emotional functioning appear to be affected.

While increased BMI usually is associated with reduced HRQoL in the general population, there is no such data published for the ACHD population [[6], [7], [8]]. A recent systematic review from 2021 confirms that the global obesity epidemic has impacted both the general public and patients with ACHD alike, suggesting not only an increase in the ACHD population, but also one that suffers from obesity-related concerns such as cardiovascular disease and lower HRQoL [9]. Furthermore, ACHD patients suffer more frequently from cardiometabolic risk factors such as hypertension, hyperlipidemia, diabetes mellitus, and more are current smokers. Regarding underweight in ACHD, a higher proportion is underweight, compared to the general population, particularly those with more severe disease [10].

Regular physical activity (PA) not only improves the cardiovascular risk profile incl. body mass index (BMI), but also improves exercise capacity and HRQoL in the general population [[11], [12], [13]]. In ACHD patients specifically, PA lowers glucose levels, improves vascular function and reduces other cardiovascular risk factors [14]. Regarding the level of PA in ACHD patients, the published data are heterogeneous with some reporting less PA than controls, while others have found no difference [[15], [16], [17], [18]].

The aim of this study was to investigate which factors are associated with optimal HRQoL in ACHD patients. We hypothesized that PA and BMI both are related to HRQoL in patients with ACHD, similar to the general population. Clinically, the results of this study are expected to emphasize the importance of routine preventive cardiology counseling of all children and adolescents with CHD.

## 2 Methods

### 2.1 Study population

For this retrospective registry study of ACHD patients. All participants were enrolled in the Swedish registry of Congenital Heart Disease (SWEDCON) in accordance with a waiver of written informed consent supported by the proper authorities. According to national guidelines, only written or oral information is required for inclusion. Individuals may choose to opt out of registration in SWEDCON, although this occurs infrequently. The study received formal approval in accordance with the declaration of Helsinki by the Regional Ethics Review Board at Lund University, Sweden (Dnr 2015/559 and 2018/434).

We included patients who had at least one outpatient visit to the ACHD clinic at Skåne University Hospital in Lund during the years 2004–2022 and had available HRQoL data (EQ-5D-3L), which patients are asked to complete at every clinic visit. If patients had multiple clinic visits, only their most recent visit was used. We included patients that could be classified based on the ESC classification. We excluded patients who did not have structural CHD that could be classified based on the ESC criteria into mild, moderate and severe [19]. The demographic data included were age, sex, weight, length, body mass index (BMI), blood pressure, ACHD severity category, cardiovascular medication, self reported PA level (none, less than 3h per week, at least 3h per week), and the results of the EQ-5D-3L. In accordance with the Centrer for Disease Control (CDC), BMI was categorized into being underweight (BMI <18.5 kg/m^2^), normal weight (BMI 18.5–24.9 kg/m^2^), overweight (BMI 25.0–29.9 kg/m^2^), or obese (BMI ≥30 kg/m^2^).

### 2.2 EQ-5D-3L

EQ-5D-3L is an HRQoL instrument that describes health in five different dimensions: mobility, self-care, regular activities, pain and anxiety/depression (https://euroqol.org). These five dimensions are then described in three severity levels, i.e. no problem, some problems and severe problems. Based upon these responses, the Time Trade-off (TTO) score is specific to the Swedish population is calculated [20]. The TTO-score is a measure how much life-time an individual is willing to trade for perfect health. A ‘’perfect’’ TTO-score in this study was the highest points one could achieve, 0.97. The visual analogue scale (EQ-VAS) is a measurement that reflects the patient's own judgement regarding their HRQoL, where “100” represents ‘the best health you can imagine’, and “0 ‘the worst health you can imagine’. Participants are then asked to point at the part of the scale that best represents their current health [21].

### 2.3 Statistical testing

For statistical analyses, continuous variables were expressed as median and inter-quartile range (IQR) as the data was not normally distributed. Categorical variables were expressed as number (percent). Group differences were analyzed using Kruskal Wallis test and Chi-Square test as appropriate. Partial correlations correcting for age, sex and ACHD severity were performed. Logistic regression analyses were carried out using a perfect TTO-score (0.97) and a VAS >90 as the outcome variable and age, sex, ACHD severity, BMI and physical activity as covariates. Odds ratios (OR; 95 % confidence interval, CI) were calculated.

To analyze important independent predictors of a perfect TTO-score, decision tree analysis was performed using the Chi-square automatic interaction detection (CHAID) algorithm. Independent variables included in the model were sex, ACHD severity category as well as BMI category. Age was entered as an influencing value.

For statistical analyses, Statistical Product and Service Solutions (SPSS, version 29, IBM, New York, USA) was used.

## 3 Results

### 3.1 Cohort characteristics based on disease severity

A total of 2671 patients from the ACHD clinic at Skåne Universsity Hospital in Lund met inclusion criteria. We excluded 202 patients that could not be categorized according to the ESC ACHD severity criteria (e.g. isolated patent foramen ovale, spontaneously closed septal defect, Kawasaki disease, innocent murmur, congenital atrioventricular block, vascular ring or coronary fistula).

Thus, the study cohort consisted of 2469 ACHD patients. 878 (25.6 %) patients had mild, 1151 (46.9 %) moderate and 329 (13.3 %) severe ACHD types. Data of the most recent visit was used for analysis.

### 3.2 ACHD complexity

There were significant differences in age and sex across all three ACHD complexity groups (Table 1). The median BMI was significantly lower in the severe ACHD category compared to the mild and moderate groups, which had a similar median BMI (p = 0.397). Patients in the mild vs moderate ACHD groups had a similar distribution of underweight, normal weight, overweight and obesity (p = 0.389). In the severe vs mild-moderate ACHD groups, there was a higher prevalence of underweight (7.3 % vs 4 %, p = 0.008), a trend towards a higher prevalence of normal weight (53.2 % vs 47.8 %, p = 0.058), and a lower prevalence of overweight (22.8 % vs 32.6 %, p < 0.001), while the obesity prevalence was not significantly different (p = 0.637). When examining clinical descriptive variables, there were no significant difference in systolic and diastolic blood pressure between the mild and moderate ACHD categories (all p > 0.1). However, systolic blood pressure was significantly lower in the severe compared to the mild and moderate ACHD group. Usage of cardiovascular medications or presence of cardiovascular symptoms increased with increasing ACHD severity category (all p < 0.001; Table 1). Concerning physical activity, with increasing ACHD severity, fewer patients were pursuing regular physical activity (Table 1).

**Table 1:** Descriptive statistic of the study population based on the severity of ACHD according to the ESC guidelines [19]. The p-values listed were created to determine overall significance across all severity categories. Values are presented as median range or percentages.

Descriptives	Mild (n = 922)	Moderate (n = 1192)	Severe (n = 355)	P(all)
Age	35 (25–50)	35 (26–48)	31 (25–39)	<0.001
Sex (female %)	516 (56 %)	522 (43.8 %)	161 (45.4 %)	<0.001
Cardiovascular medications	246 (26.7 %)	453 (38 %)	221 (62.3 %)	<0.001
Cardiovascular symptoms	178 (19.3 %)	238 (20 %)	125 (35.2 %)	<0.001
BMI	24.7 (22.0–27.0)	24.8 (22.1–28.3)	23.8 (21.5–27.2)	0.006
BMI Category				0.001
- Underweight	43 (4.7 %)	41 (3.4 %)	26 (7.3 %)	
- Normal weight	433 (47 %)	577 (48.4 %)	189 (53.2 %)	
- Overweight	308 (33.4 %)	381 (32 %)	81 (22.8 %)	
- Obese	138 (15 %)	193 (16.2 %)	59 (16.6 %)	
Systolic BP	125 (115–135)	125 (115–136)	120 (110–130)	<0.001
Diastolic BP	75 (70–80)	75 (70–80)	75 (70–80)	0.220
Physical activity				0.026
- No sport	378 (41 %)	555 (46.6 %)	174 (49 %)	
- <3 h/week	292 (31.7 %)	351 (29.4 %)	122 (34.4 %)	
- ≥3 h/week	181 (19.6 %)	217 (18.2 %)	50 (14.1 %)	
HRQoL: no problems with				
- Mobility	798 (87.0 %)	1036 (87.1 %)	279 (78.8 %)	<0.001
- Self care	886 (96.5 %)	1115 (94.1 %)	322 (91.0 %)	<0.001
- Usual activities	797 (86.8 %)	1010 (85.1 %)	258 (73.1 %)	<0.001
- Pain & discomfort	619 (67.8 %)	798 (67.4 %)	225 (64.5 %)	0.511
- Anxiety & depression	626 (68.4 %)	783 (66.5 %)	209 (59.7 %)	0.013
TTO score	0.97 (0.88–0.97)	0.97 (0.88–0.97)	0.91 (0.79–0.97)	<0.001
VAS score	88.9 (72.2–88.9)	88.9 (72.2–88.9)	79.1 (63.0–88.9)	<0.001

Abbreviations: ACHD: Adults with Congenital Heart Defects, BMI: Body Mass Index. HRQoL: Health-related quality of life, VAS-Score: Visual Analogue Scale.

In regard to HRQoL, we evaluated the five dimensions mobility, self-care, usual activities, pain and discomfort and anxiety and depression as well as the TTO score and VAS scale (Table 1). Overall, there were significant differences between the ACHD severity groups for all dimensions except for pain and discomfort (Table 1). Patients with mild-moderate ACHD severity had similar HRQoL parameters (Table 1; all p > 0.2 except self-care: p 0.01). Compared to mild-moderate ACHD, the severe group had lower levels of functioning in the domain mobility (p < 0.001), self-care (p 0.002), usual activities (p < 0.001), and anxiety and depression (p 0.006), but no significant difference in pain and discomfort was found (p = 0.268; Table 1). Likewise, the TTO and VAS scores in the severe ACHD group were significantly lower than in the mild and moderate groups (Table 1).

### 3.3 BMI and PA in relation to HRQoL

We tested whether an abnormally low or high BMI and physical activity correlate with HRQoL parameters. We therefore divided the cohort into 1. a low and normal BMI group and into 2. a normal and high BMI. We then performed partial correlations correcting for age, sex and ACHD severity category. In the low-normal BMI cohort, both PA and BMI both correlated with TTO score and all other above-named HRQoL variables (TTO: PA R 0.23, p < 0.001; BMI R 0.12, p < 0.001), but the correlations for PA were generally stronger than for BMI. BMI also correlated positively with more PA (R 0.15, p < 0.001).

We then looked at the subgroup of patients with a normal and high BMI. We found that higher HRQoL was correlated with a higher level of self-reported PA and a lower weight (TTO: PA R 0.17, p < 0.001; BMI R −0.12, p < 0.001). Similar findings were noted on the domain level (data not shown). BMI also correlated negatively with more PA (R −0.14, p < 0.001).

In a logistic regression model (Table 2), physical activity was the most important predictor of a perfect HRQoL score in all five domains (mobility, self-care, usual activities, pain & discomfort, and anxiety & depression), especially if performed for >3 h/week (OR 2.1–7.5, all p < 0.001). Other important predictors of a perfect TTO score were younger age, male sex, mild-moderate CHD (OR 1.59, p < 0.001) and being of normal weight (OR 1.44, p < 0.001; Table 2). Interestingly, underweight patients had similar HRQoL as obese patients (Table 2).

**Table 2:** Predictors of perfect HRQoL based on the TTO score and individual EQ-5D-3L domains.

	TTO perfect	Mobility	Self-Care	Usual activities	Pain & Discomfort	Anxiety & Depression
	OR (95 % CI)	OR (95 % CI)	OR (95 % CI)	OR (95 % CI)	OR (95 % CI)	OR (95 % CI)
Age	0.99 (0.98–0.99)∗∗	0.97 (0.96–0.98)∗∗	0.99 (0.98–1.00)	0.99 (0.98–1.00)∗∗	0.99 (0.98–0.99)∗∗	0.99 (0.98–1.00)∗
Male sex	1.58 (1.33–1.87)∗∗	1.88 (1.45–2.44)∗∗	1.40 (0.96–2.04)	1.76 (1.39–2.23)∗∗	1.54 (1.28–1.85)∗∗	1.39 (1.16–1.66)∗∗
ACHD category						
- Moderate	0.89 (0.74–1.07)	0.88 (0.66–1.17)	0.66 (0.42–1.03)	0.86 (0.66–1.13)	0.94 (0.77–1.15)	0.89 (0.73–1.08)
- Severe	0.60 (0.46–0.78)∗∗	0.36 (0.25–0.52)∗∗	0.41 (0.24–0.69)∗∗	0.36 (0.26–0.50)∗∗	0.77 (0.58–1.01)	0.65 (0.50–0.85)∗∗
BMI category						
- Overweight	0.81 (0.67–0.99)∗	0.79 (0.58–1.07)	1.17 (0.73–1.87)	0.79 (0.60–1.04)	0.87 (0.71–1.08)	0.77 (0.63–0.95)∗
- Obese	0.67 (0.53–0.87)∗∗	0.42 (0.30–0.57)∗∗	0.67 (0.42–1.06)	0.64 (0.47–0.88)∗∗	0.66 (0.51–0.85)∗∗	0.80 (0.62–1.03)
- Underweight	0.54 (0.34–0.84)∗∗	0.44 (0.25–0.78)∗∗	0.37 (0.18–0.73)∗∗	0.48 (0.29–0.79)∗∗	0.64 (0.41–1.00)	0.69 (0.44–1.07)
Physical Activity						
- <3 h/week	1.41 (1.16–1.71)∗∗	2.25 (1.67–3.03)∗∗	1.66 (1.09–2.52)∗	1.54 (1.18–2.00)∗∗	1.43 (1.16–1.75)∗∗	1.28 (1.05–1.56)∗
- ≥3 h/week	2.40 (1.88–3.07)∗∗	6.76 (3.61–12.65)∗∗	7.54 (2.72–20.93)∗∗	3.22 (2.10–4.93)∗∗	2.35 (1.77–3.10)∗∗	2.11 (1.61–2.76)∗∗

∗∗p < 0.005, ∗p < 0.05. Abbreviations: TTO: Trade off time, VAS: Visual Analogue Scale, ACHD: Adults with Congenital Heart Disease, BMI: Body mass Index, OR: Odds ratio, CI: Confidence interval.

### 3.4 Physical activity

**Fig. 1:**
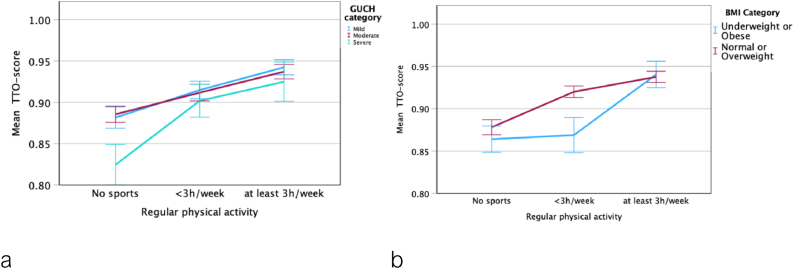
Association of HRQoL with Physical Activity and a) CHD Severity, b) BMI category. Mean TTO score with 95 % confidence interval.

We evaluated the cohort based on the amount of self-reported weekly PA. Patients who were not doing regular exercise were significantly older, more likely to be female, had a higher BMI, and a lower HRQoL than their physically active peers (Table 3). PA ≥3 h/week had a stronger effect than PA <3 h/week (Table 3). Also, HRQoL was decreased with increasing ACHD severity category. Patients with severe CHD benefitted the most, when performing some exercise (<3h vs none; Fig. 1a). Liklewise, ACHD patients with underweight and obesity alike had lower HRQoL than their normal weight or overweight peers if they were physically active, but less than 3 h/week (Fig. 1b)

**Table 3:** Descriptive statistic of the study population based on the reported amount of physical activity per week. The p-values listed were created to determine overall significance across all severity categories. Values are presented as median range or percentages.

	No sports (n = 1107)	<3 h/week (n = 765)	≥3 h/week (n = 448)	p(all)
DEMOGRAPHIC VARIABLES				
Age	38 (28–52)	34 (26–45)	29 (23–37)	<0.001
Female sex (%)	583 (52.6 %)	364 (47.6 %)	171 (38.2 %)	<0.001
BMI	25.4 (22.2–29.1)	24.3 (21.8–27.4)	24.0 (21.9–26.2)	<0.001
BMI Category				
- Underweight	48 (4.3 %)	35 (4.6 %)	15 (3.3 %)	<0.001
- Normal weight	475 (42.9 %)	392 (51.2 %)	265 (59.2 %)	
- Overweight	342 (30.9 %)	240 (31.4 %)	140 (31.3 %)	
- Obese	242 (21.9 %)	98 (12.8 %)	28 (6.3 %)	
ACHD Category				0.026
- Mild	378 (34.1 %)	292 (38.2 %)	181 (40.4 %)	
- Moderate	555 (50.1 %)	351 (45.9 %)	217 (48.4 %)	
- Severe	174 (15.7 %)	122 (15.9 %)	50 (11.2 %)	
EQ5D: No problems with				
- Mobility	857 (77.8 %)	694 (90.8 %)	435 (97.5 %)	<0.001
- Self-care	1012 (92.2 %)	730 (95.5 %)	443 (99.1 %)	<0.001
- Usual activities	863 (78.5 %)	659 (86.3 %)	420 (94.0 %)	<0.001
- Pain & discomfort	649 (59.3 %)	533 (70.1 %)	363 (81.6 %)	<0.001
- Anxiety & depression	661 (60.3 %)	514 (67.6 %)	348 (79.1 %)	<0.001
TTO-score	0.91 (0.85–0.97)	0.97 (0.88–0.97)	0.97 (0.91–0.97)	<0.001

### 3.5 Independent predictors of good health related quality of life using decision tree analyses

**Fig. 2:**
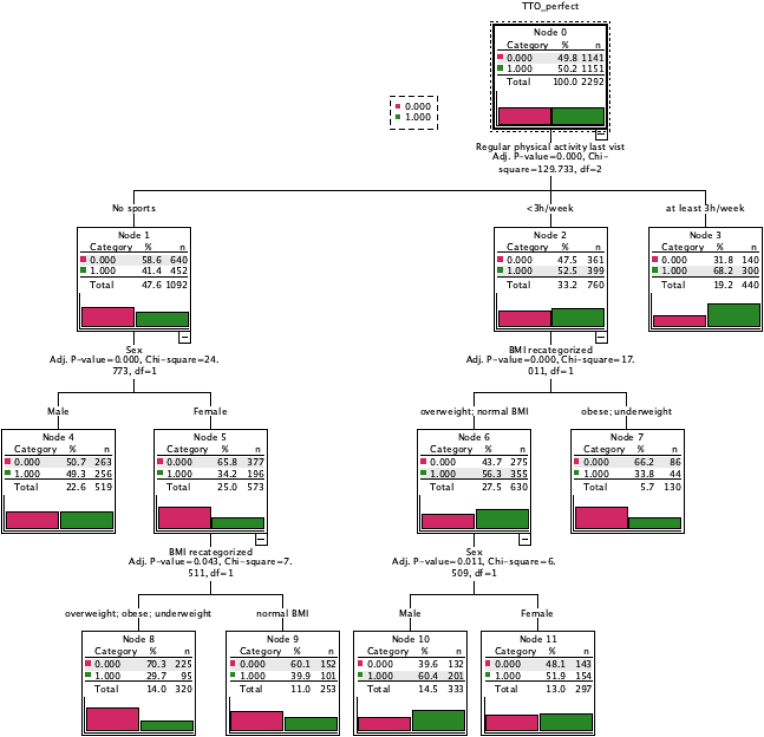
Decision tree indicating factors independently associated with a perfect TTO score (0.97) in patients with ACHD. The decision tree splits first at physical activity, indicating that this is the most important determinant of health-related quality of life defined as a perfect TTO score. The number and percentage for a perfect TTO-score are shown for each node. Abbreviations: TTO: Time-trade off. BMI: Body mass index. ACHD: Adults with congenital heart defects.

To supplement the above analyses, and identify the most important independent predictors of HRQoL, we used decision tree analyses. Only patients with available physical activity data were included (n = 2299). We included possible predictors of a perfect TTO-score (0.97). These were ACHD severity, BMI, PA and sex with age as an influencing variable. We found that 68.2 % of those that did PA ≥3h per week had perfect TTO-scores (Fig. 2). In patients who did <3 h of physical activity per week, BMI was the next important factor with the obese and underweight having lower chances of having a perfect TTO score. In those who performed some PA and had a normal BMI or overweight, male sex was associated with perfect TTO scores. Lastly, in the physically inactive group, female sex was associated with lower chances of having a perfect TTO score, especially if their weight was outside the normal range (Fig. 2).

## 4 Discussion

This large single center registry based study of ACHD patients evaluated the association between HRQoL, physical activity, and BMI. Our findings indicate that while both physical activity and BMI have an impact on HRQoL, the most important modifiable factor is physical activity. Notably, underweight as well as obese patients have worse HRQoL than their normal weight or overweight peers. In addition, non-modifiable risk factors for worse HRQoL are increasing age, female sex and severe ACHD. We demonstrated that exercise is incrementally associated with better HRQoL. In patients with severe CHD, even exercise <3 h/week appears to have a positive effect on HRQoL. Patients with underweight or obesity, however, need to execise more than their normal weight/overweight peers, in order to achieve a high HRQoL.

Over the last 50 years, a paradigm shift has developed in the field of congenital cardiology. While short term survival was the focus early on, this gradually shifted to improving long term survival and ultimately HRQoL. As most patients with CHD survive well into adulthood, traditional cardiovascular risk factors for acquired heart disease need to be addressed as well in order to achieve optimal long-term outcomes. In fact, ACHD patients with mild to moderate defects appear to be at disproportionately increased risk for acquired cardiovascular disease, even when adjusting for risk factors [3]. Thus, it appears particularly important to maintain a healthy life style and address modifiable risk factors. Unfortunately adults with CHD were oftened conditioned otherwise during childhood. Without data to support this, providers used to restrict their patients light-mindedly from exercise, hoping to reduce the risk of arrhythmias and/or heart failure [22].

Fortunately, cardiology associations from both sides of the Atlantic ocean have meanwhile published CHD-specific exercise recommendations [23]. Nonetheless, 38 % of children and adolescents with moderate-severe CHD (Tetralogy of Fallot, transposition of the great arteries, and single ventricle patients following Fontan palliation) reported being exercise restricted by their cardiologist [22]. Interestingly, patients who were restricted to only mild exertion had a higher BMI. Thus, there is a gap between expert consensus and clinical implementation that needs to be addressed urgently. As life style habits are shaped during childhood and adolescence, prevention and lifestyle counseling in congenital cardiology has to be (or become) part of every pediatric cardiology visit. Moreover, overweight CHD patients should be referred early to pediatric obesity programs early.

In addition to physicians restricting their patients from exercise, parents are prone to "protect" their child's heart. Longmuir et al. (2021) found that parental uncertainty towards PA contributes to an inactive lifestyles inspite of minimal restrictions by the medical team [24]. The authors concluded that PA should more actively be encouraged by the provider.

Parents of infants and children with CHD are faced with many uncertainties. Many parents of infants with CHD suffer from anxiety, depression and post-traumatic stress symptoms, especially if the CHD was diagnosed postnatally [25]. This uncertainty can affect parenting style and may be transferred on to the developing child. This was demonstrated by Dulfer et al. (2015) in an exercise intervention study on adolescents with Tetralogy of Fallot or Fontan physiology. The authors demonstrated that participants reported a decrease in HRQoL following the exercise program when their parents showed poorer overall mental health themselves [26]. By contrast, positive parenting practices may be protective against anxiety and other mental health symptoms in adulthood, which again are risk factrors for acquired cardiovascular disease [27,28].

In our study, PA is associated with better HRQoL, as has been shown previously by others in both patients with CHD and the general population [11,[29], [30], [31]]. BMI was also associated with HRQoL, but appears ot be a secondary factor (Fig. 2). Interestingly, underweight and obesity had a similar effect on HRQoL in our study.

In underweight patients with and without CHD, HRQoL is diminished as we and others have shown. [[32], [33], [34]]. In our study underweight ACHD patients had a lower HRQoL than the overweight and normal categories. The severe CHD group was at highest risk for underweight. This was consistent with prior studies and also in comparison to population data provided by the Public Health Agency of Sweden [[35], [36], [37]]. Reasons for this could be malnutrition due to diminished appetite, poor diet, protein losing enteropathy, increased metabolic demands, anemia, or congestive heart failure.

Overweight and obesity in the general population as well as in chronically ill patients is associated with lower HRQoL, even though in our study only obesity impacted HRQoL significantly. [33,38,39]. Follwoing weight loss, statistically significant enhancements in HRQoL have been reported in the general population [40]. Similar studies in ACHD patients have not been performed.

We also discovered that male sex had higher odds of having a perfect HRQoL score in our population, which is in agreement with prior studies. [[41], [42], [43]]. Age has also been identified as a strong predictor of HRQoL by us and in the general population [44,45].

### 4.1 Limitations

This study identified several important associations, but due to the nature of a registry study, causality could not be evaluated. The EQ-5D-3L is an easy to perform but crude measure of HRQoL. Nonetheless, all associations were highly significant. Lastly, physical activity levels were self-reported, so that recall bias as well as different views on what is considered "physical activity" may have affected our results. In addition, the study lacks supporting objective exercise data and is a single-center study.

## 5 Conclusion

Our findings show that physically active ACHD patients with a normal BMI have a high HRQoL, particularly if they suffer from mild-moderate disease complexity, are male and of young age. Thus, individualized care regarding physical activity and discussions regarding importance of maintaining a normal body weight should be brought up during routine pediatric cardiology visits, starting during childhood and adolescence. In the future, structured life-style interventions (e.g. exercise programs and nutrition counseling) for children, adolescents and adults with CHD should be implemented. The effect of such formal programs on HRQoL and BMI should be assessed longitudinally.

## CRediT authorship contribution statement

**Constance G. Weismann:** Writing – original draft, Visualization, Supervision, Methodology, Investigation, Formal analysis, Conceptualization. **Frishta Jafar:** Writing – original draft, Investigation, Formal analysis, Data curation. **Joanna Hlebowicz:** Writing – review & editing, Resources, Data curation.

## Declaration of competing interest

The authors declare that they have no known competing financial interests or personal relationships that could have appeared to influence the work reported in this paper.
